# 

**Published:** 1915-09-25

**Authors:** 


### The American Red Cross Society.

For financial reasons the American Red Cross Society proposes to withdraw the units from Europe on October 1. The Society feels that, having given the assistance of its personnel for one year, it can now render a better service by continuing simply to furnish surgical, hospital, and medical supplies to the belligerent nations. They will do this probably for the entire duration of the war, by withdrawing the units, and thus saving the expense of keeping such personnel in Europe. The personnel sent to Europe comprised 63 surgeons, 217 nurses, and twelve sanitary commissions. The American Women's War Hospital at Oldway House, Paignton, will not be closed, nor will the continuity of the work be in any way interrupted by the decision of the American Red Cross. Two of the American Red Cross nursing units have worked at Paignton for many months, and, immediately on receiving an official intimation of their withdrawal, the American Women's War Relief Fund took the necessary steps with regard to their hospital. An American chief surgeon has been appointed, and, as heretofore, the staff will comprise both English and American sisters.

### A Token of Gratitude and Fraternity.

THE French colony in Milan, as an act of gratitude and fraternity to their neighbours, has given a hospital for wounded Italians in that city. The hospital, which consists of three large wards with forty beds each, was opened on September 14 by the French Consul-General.

### Latest Installations in Hospital Equipment.

### WHAT OTHERS ARE DOING.

***** We desire information calculated to keep this column up to date in the interest of institutional workers generally. Secretaries and other executive officers are invited to forward particulars of new installations in any department. In this way a file will be available for all hospital authorities contemplating improvements and anxious to know what other institutions have found effective and modern.**

AUXILIARY HOSPITALS AND HUTS.

MESSRS. HUMPHREYS, of Knightsbridge, London, have recently erected Queen Mary's Convalescent Auxiliary Hospital Building at Roehampton and several hundred huts for the Admiralty and War Office. They also keep in stock a number of hospitals and huts for inspection by members of the medical profession.

### Publishers' Notices.

A SECOND and entirely revised and enlarged edition of "Diseases of the Nervous System," by H. Campbell Thomson, M.D., F.R.C.P., physician to the department for diseases of the nervous system and lecturer on neurology, Middlesex Hospital, is announced by Messrs. Cassell. The investigations into syphilis of the nervous system, which have led to its division into interstitial and parenchymatous forms, have necessitated a reconstruction of this part of the book. Tabes and general paralysis now fall into their places according to their etiology. New chapters have been included on the general functions of the brain, the sympathetic system, and the paths of infection of the central nervous system, and the section dealing with the classification of neurons has been enlarged. The illustrations have also been added to.

MESSRS. HENRY FROWDE, HODDER AND STOUGHTON (Oxford Press Warehouse, Falcon Square, London, E.C.) have notified their intention to publish a companion to the R.A.M.C. Training Book, entitled "The Stretcher-Bearer," illustrating the stretcher-bearer drill and the handling and carrying of wounded, by Georges M. Dupuy, M.D., containing 168 pages, with 138 illustrations (price 2s. net). The book is intended to form a companion to the R.A.M.C. Training Book, showing the different evolutions of the stretcher-bearers by photographs illustrating the execution of each special command. It is specially intended for the assistance of those who have had no experience in military or ambulance training, to enable them to form a clear idea of a command by seeing the order actually carried out.

### Training in Dispensing Work.

SPECIAL day and evening courses have been arranged at the South-Western Polytechnic Institute, Manresa Road, Chelsea, for women students desiring to pass the assistants' examination of the Society of Apothecaries, which qualifies them to act as dispensers to a medical man, and also, under certain conditions, in a hospital. The day course includes book-keeping and typewriting, in addition to chemistry, pharmacy, and materia medica. A good knowledge of the two former subjects enables women to obtain better and more lucrative employment. Particulars of fees and hours may be obtained from the Secretary of the Institute.

RATES OF SUBSCRIPTION: Twelve months, 6/6; Six months, 4/-; Three months, 2/-, post free to any address in the United Kingdom. Abroad, Twelve months, 8/8; Six months, 5/-; Three months, 2/6, post free.—Address The Subscription Dept., "THE HOSPITAL," The Hospital Building, 28/29 Southampton Street, Strand, London, W.C.

By Sir ARBUTHNOT LANE, Bart., M.S.

OPERATIVE TREATMENT OF FRACTURES.

*SECOND EDITION. With* 226 *Illustrations.* 10s.

THE MEDICAL PUBLISHING COMPANY, LTD., 23 Bartholomew Close, E.C.

THE OPERATIVE TREATMENT OF CHRONIC INTESTINAL STASIS.

With additional Chapters by Drs. JAMES MACKENZIE, JORDAN and MUTCH.

*THIRD EDITION. With* 102 *Illustrations.* 10s. 6d. *net.*

J. NISBET & CO., LTD., 22 Berners Street, W.

CLEFT PALATE AND HARE-LIP.

With additional Chapters by Mr. CORTLANDT MACMAHON and Mr. WARWICK JAMES.

*THIRD EDITION. With* 58 *Illustrations.* 10s.

THE MEDICAL PUBLISHING COMPANY, LTD., 23 Bartholomew Close, E.C.

